# Minor Surgical Gynæcology

**Published:** 1885-08

**Authors:** 


**Minor Surgical Gynæcology.** A Treatise of Uterine Diagnosis and the Lesser Technicalities of Gynæcological Practice, including general rules for gynæcological operations, and the operations for lacerated cervix and perineum, and prolapsus of uterus and vagina. For the use of the advanced student and general practitioner. By PAUL F. MUNDÉ, M.D. Second edition; revised and enlarged. With three hundred and twenty-one illustrations. New York: William Wood & Co., 56 and 58 Lafayette Place. 1885.

This second edition of Mundé's Minor Gynæcology needs no introduction from us to the profession. The work is prepared for those whose opportunities have not furnished the special training or minute details in this specialty, and Dr. Mundé, through long years of teaching, has ascertained the errors of the members of his classes, and in this work aims to provide the profession with accurate information to supply deficiencies which he has detected through his instructions. The work does not contain a detailed account of larger operations. Its title, "Minor Surgical Gynæcology," aptly gives its scope and its object. The work is a much needed one. Dr. Mundé is just the man to write it, and the publishers have done the profession a special favor in furnishing it.

